# The correlation and gut microbial characteristics in the whole spectrum of Alzheimer’s disease: a systematic review and meta-analysis

**DOI:** 10.3389/fnins.2026.1775002

**Published:** 2026-03-04

**Authors:** Zhao Xiaoyi, Li Haixiao, Ren Houjian, Zhang Wenya, Fan Jie, Song Han, Wang Defeng, Wang Zhen, Cao Jingrong

**Affiliations:** 1 Department of Clinical Laboratory, Xiongan Xuanwu Hospital, Xiong’an New Area, Hebei, China; 2 Xiongan Magic Medical Laboratory, Xiong’an New Area, Hebei, China; 3 School of Medicine, Hebei University of Engineering, Handan, Hebei, China; 4 School of Basic Medicine, Hebei Medical University, Shijiazhuang, Hebei, China; 5 Affiliated Hospital of Hebei University of Engineering, Handan, Hebei, China; 6 Department of Clinical Laboratory, Xuanwu Hospital, Capital Medical University, Beijing, China

**Keywords:** Alzheimer’s disease, gut microbiota, healthy controls, meta-analysis, mild cognitive impairment

## Abstract

**Background:**

Gut dysbiosis is hypothesized to be a potential pathological mechanism in patients across the Alzheimer’s disease (AD) spectrum. Nevertheless, despite growing interest, existing findings remain largely inconsistent.

**Purpose:**

This systematic review and meta-analysis aimed to compare the composition of gut microbiota (GM) between patients with mild cognitive impairment (MCI) or AD and healthy controls (HC).

**Methods:**

PubMed, Embase, MEDLINE and Web of science were searched from January 2022 to November 2025. Eligible studies included observational studies and pre-intervention arms of interventional trials reporting GM abundance in AD spectrum patients vs. HC. Two reviewers independently screened articles, extracted data, and assessed bias risk. Effect sizes were pooled using an inverse-variance weighted random-effects model.

**Results:**

Twenty studies (1,025 HC and 456 AD spectrum patients) were analyzed. AD patients demonstrated reduced GM diversity vs. HC cohort. The abundances of *Megamonas* and *Bacteroides* were elevated in AD patients, while *Firmicutes* and *Proteobacteria* were reduced. When stratified by clinical stage, *Fusobacteria* and *Lactobacillus* abundances showed gradient shift from MCI to AD.

**Conclusion:**

Individuals within the AD spectrum exhibit altered GM abundance, with these differences influenced by clinical stage. The present study did not identify any significant trends; it reports only findings that have been statistically substantiated.

## Introduction

Alzheimer’s disease (AD) is characterized by two hallmark pathologies: *β*-amyloid (Aβ) plaque deposition and neurofibrillary tangles of hyperphosphorylated tau protein (Scheltens et al., 2021; Dissanayaka et al., 2024). However, its pathogenesis remains unclear, with recent evidence highlighting the gut microbiota (GM) as a potential contributor to AD development or progression (Liu et al., 2020; Jiang et al., 2017; Grabrucker et al., 2023; Zhuang et al., 2018). The associations of the GM with updated biomarker categories of AD pathogenesis are described (2024). Current findings of the GM characteristics in the whole spectrum of AD are summarized.

The GM is a complex ecosystem consisting of over 100 trillion symbiotic microbial cells that support human physiology and development (Doifode et al., 2021). A growing body of research points to an interplay between the gastrointestinal tract and central nervous system (CNS) via the gut-brain axis: (a) Neurochemical pathways: GM synthesizes/releases neurotransmitters (e.g., serotonin, acetylcholine) and metabolites (e.g., short-chain fatty acids [SCFAs], tryptophan) that cross the blood–brain barrier (BBB) to modulate neural activity (Dicks, 2022; Li et al., 2023). (b) Neural pathways: The enteric nervous system (ENS) connects to the CNS via the vagus nerve and autonomic nervous system (Bonaz et al., 2018). (c) Immune pathways: GM regulates peripheral inflammation by producing pro-inflammatory cytokines (e.g., IL-1, IL-6, TNF-*α*) (Fröhlich et al., 2016; White et al., 2025).

Recently, GM disruption contributes to gastrointestinal disorders and systemic physiological alterations through the release of abnormal microbial metabolites. Animal studies indicate that GM dysbiosis is associated with the onset of AD (Grabrucker et al., 2023; Chen et al., 2022; Chen et al., 2024). Preclinical and clinical studies suggest gut dysbiosis disrupts gut-brain axis communication, contributing to AD (Nguyen et al., 2023; Ullah et al., 2023). Probiotics have also been shown to improve cognitive function in AD spectrumpatients (Fei et al., 2023; Kim et al., 2021). Other conditions may also be alleviated through probiotic supplementation, including psoriasis (Buhaș et al., 2023) and chronic spontaneous urticaria (Atefi et al., 2022).

However, GM composition varies by geography: For example, Zhuang et al. (China) reported increased *Bacteroidetes* and decreased *Actinobacteriain* in AD patients (Zhuang et al., 2018). while U.S. studies yielded contradictory reports (Vogt et al., 2017). Additionally, GM dysbiosis severity may differ between MCI and AD—most studies find reduced diversity in AD vs. MCI, but some report no differences (Guo et al., 2021; Zhao et al., 2025; Lwere et al., 2025; Wanapaisan et al., 2022).

To address these gaps, this meta-analysis: (1) compares GM diversity/abundance between AD spectrum patients and HC; (2) investigates the influence of clinical stage (MCI vs. AD) on GM alterations.

## Methods

### PICOD and research question

1. Population: patients with AD and MCI;
2. Intervention: Analysis of the gut microbiota in patients with AD, MCI, and systemically healthy subjects;
3. Comparators: Systemically healthy subjects;
4. Outcomes: Observation of changes in AD, MCI and systemic healthy gut microbiota;
5. Design: Case–control studies.

The research question was: Are there significant difference in MDA levels in the saliva and blood of OC patients compared to the control group?

### Literature search

In accordance with the Preferred Reporting Items for Systematic Reviews and Meta-Analyses (PRISMA) guidelines (Dubois et al., 2016) a comprehensive search was conducted across the PubMed, MEDLINE, Embase, and Web of Science databases from January 2022 to November 2025. However, in order to ensure comprehensiveness and account for delays in online publication, a screening criterion of online publication dates or search dates recorded in the databases on or after 1 January 2022 was applied. Consequently, a small number of publications with a print year of 2021 but an online publication date that met the criteria were also included. A comprehensive search strategy was employed, encompassing search terms related to AD, MCI, and gut microbiota (Supplementary material 1).

### Eligibility criteria

Two reviewers independently screened the full-text articles for eligibility. The inclusion criteria were as follows: (1) Peer-reviewed original articles (English); (2) Comparative design (AD spectrum vs. HC); (3) GM profiles from stool samples; (4) Baseline data only (for interventional studies); (5) GM taxa investigated in ≥3 studies; (6) Sufficient statistical data for effect size calculation.

The exclusion criteria were as follows: (1) Review and non-literature (patent, conference abstract, preprint, erratum or chapter); (2) Animal studies; (3) Case reports.

### Outcome measures

The primary outcomes encompassed GM diversity (including *α* diversity and *β* diversity) and disparities in GM abundance between patients with AD spectrum and HC. The secondary outcomes consisted of the effects of different countries and clinical stages on GM abundance.

### Data extraction & bias assessment

Two reviewers extracted data (participant demographics, GM indices) and assessed bias using Risk of Bias Assessment Tool for Nonrandomized Studies (RoBANS) (Kim et al., 2013) (domains: selection, confounding, exposure measurement, blinding, incomplete data, selective reporting). Disagreements were resolved via consensus.

### Statistical analysis

Effect sizes (Hedges’ g) were calculated using Comprehensive Meta-Analysis Version 3 software (Biostat Inc., Englewood, NJ, USA). Assuming non-substantial deviations from Gaussian distributions, we estimated means and standard deviations from medians, maxima, and minima using conversion formulas from Hozo et al. (2005). Heterogeneity was assessed via Q-statistic and I^2^ metric. Outliers were identified using 95% confidence interval (CI) thresholds (Noma et al., 2020; Viechtbauer and Cheung, 2010). Publication bias was quantitatively evaluated using Begg’s test, Egger’s test, and trim-and-fill (Begg and Mazumdar, 1994; Egger et al., 1997). Significance was set at *p* < 0.05.

## Results

### Study selection

**Figure 1:**
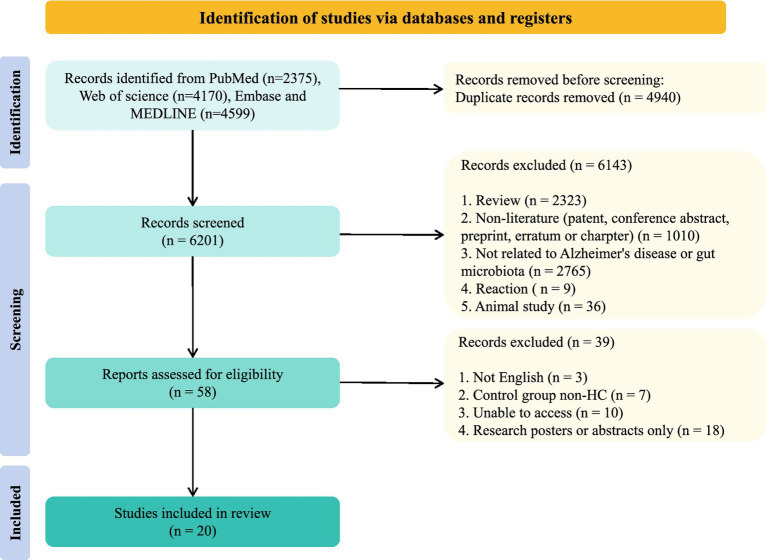
Flow diagram of selected studies.

11,141 articles were identified; 20 articles were included (Figure 1). Studies were conducted across 9 countries [Kazakhstan (Kaiyrlykyzy et al., 2022), South Korea (Kim et al., 2023; Kim et al., 2024), Japan (Yamashiro et al., 2024), Thailand (Wanapaisan et al., 2022), Turkey (Yıldırım et al., 2022), Uganda (Lwere et al., 2025), Spain (Cabrera et al., 2025; Mateo et al., 2024), China (Guo et al., 2021; Zhao et al., 2025; Chen et al., 2024; Fan et al., 2025; Fan et al., 2023; Hsu et al., 2025; Sheng et al., 2022; Zhu et al., 2022), U.S. (Kazen et al., 2025; McLeod et al., 2023; Sepúlveda-Rivera et al., 2025)] with 1,025 HC and 456 AD spectrum patients (AD = 198, MCI = 244, aMCI = 14) (Table 1).

**Table 1:** Characteristics of each study included in the meta-analysis.

Study	Country	Healthy control (HC)					Stage	AD spectrum					Dietary check
		*N*	Sex (F/M)	Age	BMI	DM^a^		*N*	Sex (F/M)	Age	BMI	DM^a^	
Cabrera et al. (2025)	Spain	38	19/19	68.21 ± 5.99	26.89 ± 4.22	5 (13.2)	AD	31	15/16	74.61 ± 5.60	25.33 ± 3.58	1 (3.2)	Yes
							MCI	30	13/17	72.13 ± 5.18	25.29 ± 2.88	5 (17.9)	
Chen et al. (2024)	China	23	NR	NR	NR	NR	AD	41	NR	NR	NR	NR	NR
Fan et al. (2025)	China	320	209/111	66.8 ± 6.8	24.7 ± 3.4	54 (18)	MCI	119	68/51	73.7 ± 7.3	24.3 ± 4.0	31 (28)	NR
Fan et al. (2023)	China	65	34/31	74.2 ± 6.1	24.4 ± 3.1	13 (20)	MCI	31	15/16	73.9 ± 6.7	24.8 ± 3.1	11 (35)	NR
Guo et al. (2021)	China	18	4/14	64.2 ± 4.7	21.1 ± 2.2	NR	AD	18	16/2	63.5 ± 4.7	21.9 ± 2.4	NR	NR
							MCI	20	16/4	64.5 ± 4.5	21.8 ± 2.0	NR	
Hsu et al. (2025)	China	54	40/14	74.2 ± 7.8	23.8 ± 4.1	NR	MCI	70	50/20	76.5 ± 7.2	23.9 ± 4.3	NR	Yes
							AD	60	42/18	79.3 ± 7.5	24.1 ± 4.5		
Kaiyrlykyzy et al. (2022)	Kazakhstan	43	35/8	68 [62, 74]^b^	27.4 [24.4, 30.1]^b^	12 (27.9)	AD	41	30/11	68 [62, 74]^b^	22.7 [21.8, 25]^b^	9 (22)	NR
Kazen et al. (2025)	U.S.	10	7/3	70.70 ± 6.13	26.06 ± 4.33	NR	MCI^c^	14	7/7	73.21 ± 6.14	26.68 ± 4.76	NR	NR
Kim et al. (2023)	KR	40	38/2	66.4 ± 5.8	23.6 ± 3.0	NR	MCI	40	34/6	69.5 ± 4.8	23.9 ± 2.2	NR	NR
Kim et al. (2024)	South Korea	17	10/7	70.82 ± 7.63	24.14 [23.01, 26.3]^b^	6 (35.29)	MCI	24	12/12	73.04 ± 6.56	22.69 [21.29, 23.72]^b^	6 (25)	NR
Lwere et al. (2025)	Uganda	13	9/4	70 [69, 71]	32.3 [28.7, 38.4]^b^	0	AD	77	63/14	76 [70, 83]^b^	28.0 [23.5, 34.3]^b^	1 (1.3)	Yes
							MCI	14	11/3	76.5 [68, 83]^b^	25.6 [21.6, 30.3]^b^		
Mateo et al. (2024)	Spain	25	12/13	70.6 ± 4.9	26.6 ± 3.2	NR	AD	25	13/12	73.0 ± 5.0	24.7 ± 3.6	NR	Yes
McLeod et al. (2023)	U.S.	30	28/2	64.9 ± 5.7	36.4 ± 4.1	NR	MCI	30	28/2	64.7 ± 5.7	36.1 ± 3.8	NR	NR
Sepúlveda-Rivera et al. (2025)	U.S.	50	36/14	NR	NR	NR	AD	50	35/15	NR	NR	NR	NR
Sheng et al. (2022)	China	34	26/8	66.91 ± 5.28	23.78 ± 2.90	5 (14.71)	AD	32	22/10	68.44 ± 5.35	24.42 ± 2.74	1 (3.13)	NR
Wanapaisan et al., 2022	Thailand	20	12/8	69.4 ± 6.2	23.8 ± 2.4	8 (40)	MCI	12	6/6	71.3 ± 4.0	24.8 ± 2.2	6 (50)	Yes
							AD	20	11/9	72.8 ± 5.6	22.8 ± 4.1	6 (30)	
Yamashiro et al. (2024)	Japan	19	12/7	78.5 ± 6.0	22.4 ± 2.3	3 (15.8)	MCI	19	13/6	81.1 ± 6.5	20.4 ± 4.8	2 (10.5)	NR
							AD	18	10/8	82.4 ± 4.5	21.2 ± 2.1	6 (33.3)	
Yıldırım et al. (2022)	Turkey	51	23/28	67 ± 5.3	NR	NR	MCI	27	11/16	69.2 ± 6.4	NR	NR	NR
Zhao et al. (2025)	China	61	36/25	62.39 ± 6.92	23.71 ± 2.65	6 (9.8)	MCI	75	41/34	64.48 ± 7.91	23.12 ± 2.81	10 (13.3)	NR
							AD	30	23/7	65.47 ± 7.99	21.38 ± 2.34	5 (16.7)	
Zhu et al. (2022)	China	94	58/36	74.3 ± 10.6	NR	13 (13.8)	MCI	125	76/49	75.4 ± 7.1	NR	20 (16)	NR
							AD	83	53/30	71.8 ± 8.3	NR	10 (12.2)	

MCI, mild cognitive impairment; AD, Alzheimer’s disease; BMI, Body Mass Index; DM, diabetes mellitus; M, male; F, female; NR, not reported; QIIME, Quantitative Insights Into Microbial Ecology; RDP, Ribosomal Database Project. ^a^DM was presented as *n* (%); ^b^Age and ^b^BMI were presented as median [interquartile range]; ^c^Amnestic MCI; ^d^The patients consisted of MCI (*n* = 15) and AD (*n* = 14).

### GM diversity

#### α diversity

**Figure 2:**
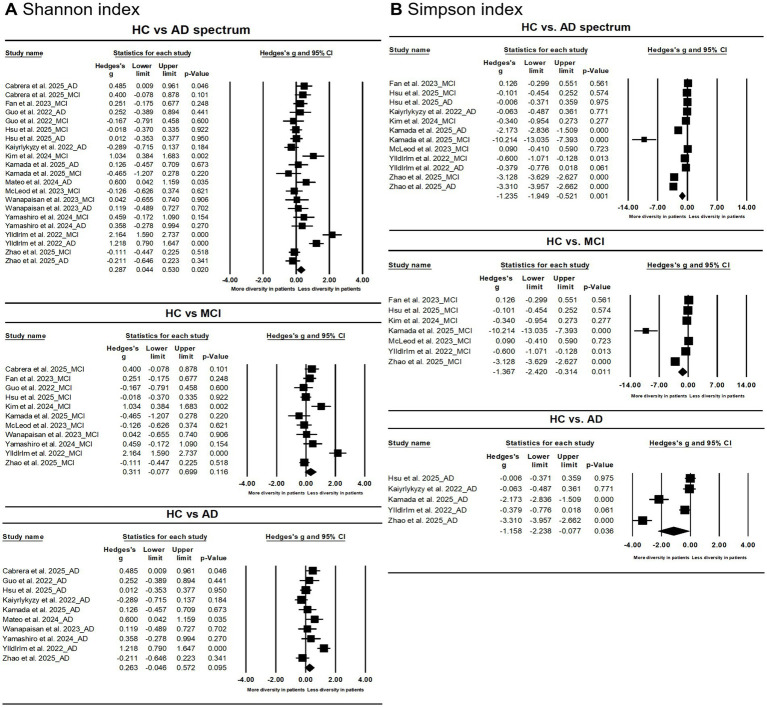
Forest plots of Shannon index **(A)** and Simpson index **(B)** in the comparisons between healthy controls (HC) and Alzheimer’s disease (AD) spectrum. Patients with AD spectrum consisted of mild cognitive impairments (MCI) and AD.

The results demonstrated that AD spectrum patients demonstrated reduced α diversity as indexed by Shannon index (Hedges’s g = 0.287; 95% CI = 0.044 to 0.530; *p* = 0.020; *n* = 21) and Simpson indices (Hedges’s g = −1.235; 95% CI = −1.949 to −0.521; *p* = 0.001; *n* = 12) vs. HC (Figure 2). Stratification showed only the Simpson index differed between MCI/AD and HC.

#### β diversity

Among all the included articles except for four studies (Wanapaisan et al., 2022; Mateo et al., 2024; Kazen et al., 2025; Sepúlveda-Rivera et al., 2025), sixteen indicators of β diversity were assessed. Results were inconsistent—weighted UniFrac distances showed significant differences in 3 studies (Guo et al., 2021; Zhao et al., 2025; Lwere et al., 2025) but not 5 others (Kaiyrlykyzy et al., 2022; Yamashiro et al., 2024; Fan et al., 2025; Fan et al., 2023; McLeod et al., 2023); unweighted UniFrac showed significance in 1 study (Yamashiro et al., 2024) but not 3 others (Fan et al., 2025; Fan et al., 2023; McLeod et al., 2023) (Table 2).

**Table 2:** Summary of β diversity assessments in the included studies.

Study	β diversity	Findings	*p*-value
Cabrera et al. (2025)	PCoA based on Bray–Curtis dissimilarity	No significant difference among HC, MCI and AD	NR
Chen et al. (2024)	PCoA based on Bray–Curtis dissimilarity	A significant difference in gut microbial composition among AD, MCI and HC	*p* = 0.0013
Fan et al. (2025)	PCoA of Weighted UniFrac distances	No significant difference between MCI and HC	NR
	PCoA of Unweighted UniFrac distances	No significant difference between MCI and HC	NR
	PCoA based on Bray–Curtis dissimilarity	No significant difference between MCI and HC	NR
Fan et al. (2023)	PCoA of Weighted UniFrac distances	No significant difference between MCI and HC	NR
	PCoA of Unweighted UniFrac distances	No significant difference between MCI and HC	NR
Guo et al. (2021)	PCoA of Weighted UniFrac distances	A significant difference in gut microbial composition between AD and HC	*p* = 0.016
		A significant difference in gut microbial composition between MCI and HC	*p* = 0.017
	PCoA based on Bray–Curtis dissimilarity	A significant difference in gut microbial composition between AD and HC	p < 0.001
		A significant difference in gut microbial composition between MCI and HC	p < 0.001
Hsu et al. (2025)	PCoA based on Bray–Curtis dissimilarity	A moderate differences in gut microbial composition between MCI and HC	NR
		A significant difference in gut microbial composition between MCI and AD	NR
Kaiyrlykyzy et al. (2022)	PCoA of Weighted UniFrac distances	No significant difference between AD and HC	*p* = 0.2313
Kim et al. (2023)	Unweighted UniFrac distance matrix	No significant difference between MCI and HC	
	Weighted UniFrac distance matrix	No significant difference between MCI and HC	
	Jaccard distance matrix	No significant difference between MCI and HC	
	Bray Curtis distance matrix	No significant difference between MCI and HC	
Kim et al. (2024)	PCoA based on Bray–Curtis dissimilarity	A significant difference in gut microbial composition between MCI and HC	*p* = 0.018
	PCoA of Generalized UniFrac distances	A slight difference in gut microbial composition between MCI and HC	*p* = 0.034
Lwere et al. (2025)	PCoA of Weighted UniFrac distances	A significant difference in gut microbial composition among AD, MCI and HC	NR
		A slight difference in gut microbial composition between MCI and HC	NR
	PCoA based on Bray–Curtis dissimilarity	A significant difference in gut microbial composition among AD, MCI and HC	NR
McLeod et al. (2023)	PCoA of Unweighted UniFrac distances	No significant difference between MCI and HC	*p* = 0.38
	PCoA of Weighted UniFrac distances	No significant difference between MCI and HC	*p* = 0.46
	PCoA based on Jaccard index	No significant difference between MCI and HC	p = 0.81
	PCoA based on Bray-Curtis index	No significant difference between MCI and HC	*p* = 0.39
Sheng et al. (2022)	NMDS based on ASV distribution	No significant difference between AD and HC	NR
	PCoA based on the distribution of ASVs	No significant difference between AD and HC	NR
Yamashiro et al. (2024)	PCoA of Unweighted UniFrac distances	A significant difference in gut microbial composition among AD, MCI and HC	p = 0.03
	PCoA of Weighted UniFrac distances	No significant among AD, MCI and HC	*p* = 0.155
Yıldırım et al. (2022)	PCoA based on Bray-Curtis distance	A significant difference in gut microbial composition among AD, MCI and HC	NR
	PCoA based on Jaccard distance	A significant difference in gut microbial composition among AD, MCI and HC	NR
Zhao et al. (2025)	PCoA based on Bray-Curtis distance	A significant difference in gut microbial composition between AD and HC	*p* = 0.0015
		A significant difference in gut microbial composition between AD and MCI	*p* = 0.00432
	PCoA of Weighted UniFrac distances	A significant difference in gut microbial composition between AD and HC	*p* = 0.00337
		A significant difference in gut microbial composition between AD and MCI	*p* = 0.009
		No significant difference between MCI and HC	NR
Zhu et al. (2022)	PCA based on Bray-Curtis distance	No significant difference among HC, MCI and AD	NR
	PCA of Unweighted UniFrac distances	No significant difference among HC, MCI and AD	NR
	PCA of Weighted UniFrac distances	No significant difference among HC, MCI and AD	NR

HC, healthy control; MCI, mild cognitive impairment; AD, Alzheimer’s disease; NMDS, Non-metric multidimensional scaling; PCoA, Principal Coordinate Analysis; PCA, Principal Component Analysis.

### GM abundance

**Figure 3:**
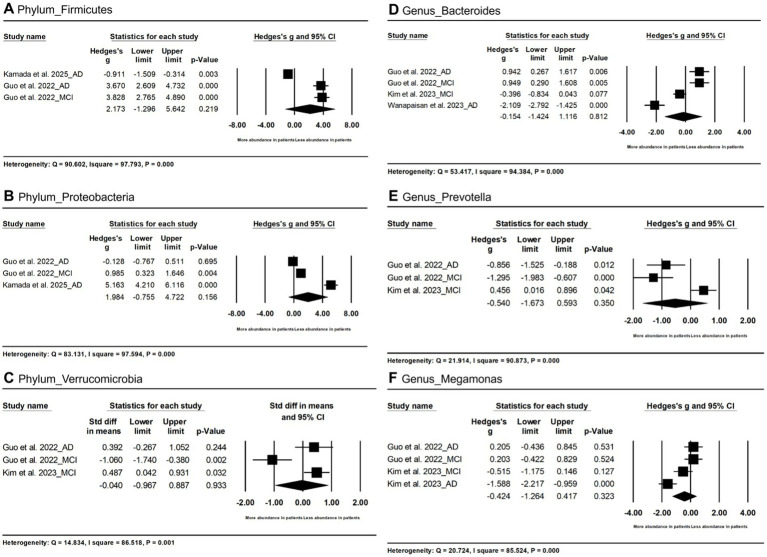
Forest plots of alterations of gut microbiota at the phylum level and genus level, including *Firmicutes* **(A)**, *Proteobacteria* **(B)**, *Verrucomicrobia* **(C)**, *Bacteroides* **(D)**, *Prevotella* **(E)**, and *Megamonas* **(F)**. AD, Alzheimer’s disease; MCI, mild cognitive impairments.

In terms of the phylum level (Figures 3A–C), no significant differences in *Firmicutes* (Hedges’s g = −2.173; 95% CI = −1.296 to 5.642; *p* = 0.219; *n* = 3), *Proteobacteria* (Hedges’s g = 1.984; 95% CI = −0.755 to 4.722; *p* = 0.156; n = 3) and *Verrucomicrobia* (Hedges’s g = −0.040; 95% CI = −0.967 to 0.887; *p* = 0.933; n = 3) between AD spectrum and HC due to high heterogeneity.

In terms of the genus level (Figures 3D–F), no significant differences in *Bacteroides* (Hedges’s g = −0.154; 95% CI = −1.424 to 1.116; *p* = 0.812; n = 4), *Prevotella* (Hedges’s g = −0.540; 95% CI = −1.673 to 0.593; *p* = 0.350; n = 3) and *Megamonas* (Hedges’s g = −0.424; 95% CI = −1.264 to 0.417; *p* = 0.323; n = 4) in AD spectrum patients vs. HC.

### Secondary outcome: clinical stage effects

The abundance of *Proteobacteria* was decreased in MCI vs. HC (Hedges’s g = 0.985, 95% CI = 0.323 to 1.646, *p* = 0.004; n = 2) but not AD vs. HC (Hedges’s g = 2.505, 95% CI = −2.680 to 7.690, *p* = 0.344; n = 2). Firmicutes showed a trend toward reduction in AD vs. HC (g = 2.891, *p* < 0.001) In addition, we found a trend toward decreased abundance of *Firmicutes* in the patients with AD (Hedges’s g = 2.891, 95% CI = −2.216 to 7.998, p < 0.001; n = 2) (Table 3).

**Table 3:** Summary of effect sizes with 95% CI when clinical stage is considered as a moderator.

Microbial species	HC vs AD			HC vs MCI		
	Hedges’s g	95% CI	*p*	Hedges’s g	95% CI	*p*
Phylum_Firmicutes	2.891	[−2.216, 7.998]	< 0.001	−0.039	[−0.663, 0.584]	0.902
Phylum_Proteobacteria	2.505	[−2.680, 7.690]	0.344	0.985	[0.323, 1.646]	0.004
Genus_Bacteroides	−0.583	[−3.573, 2.407]	0.702	0.253	[−1.063, 1.570]	0.706
Genus_Megamonas	−0.693	[−2.450, 1.064]	0.440	−0.147	[−0.851, 0.556]	0.681

The effect sizes were reported only when the number of investigations ≧ 2 in both diagnoses.

### Bias & heterogeneity

**Figure 4:**
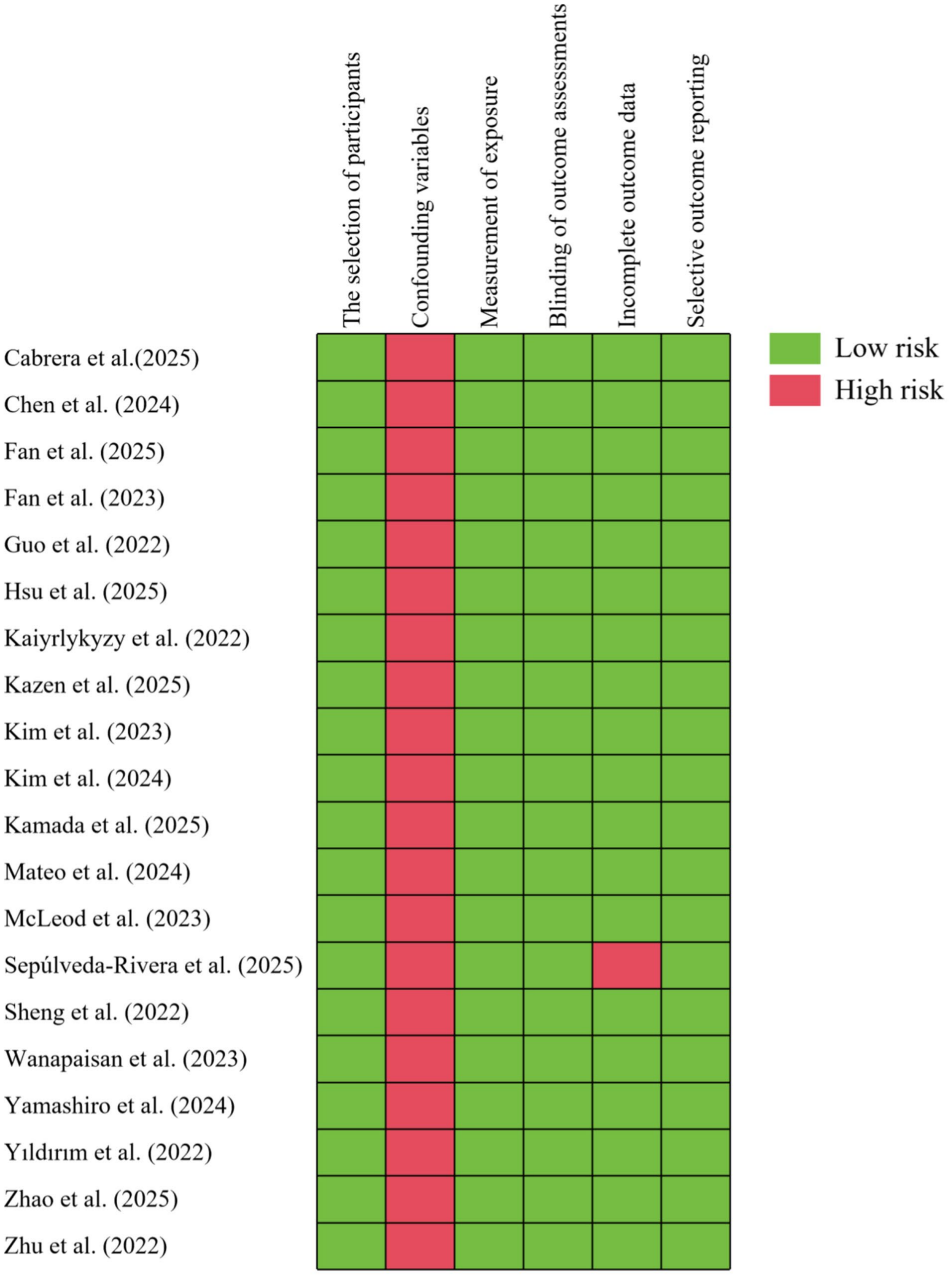
Risk of bias assessment of 20 included studies using RoBANS. RoBANS, Risk of Bias Assessment tool for Non-randomized Studies.

The quality of the included studies is summarized in Figure 4. It was determined that each study was to be categorized as low risk, in accordance with the evaluation criteria outlined in the study’s methodology. With regard to data completeness, all studies except one were rated as low risk. However, concerning the confounding variables criterion, all studies were rated as high risk of bias due to the potential influence of confounding factors, such as body mass index, diabetes, and diet, on brain grey matter structure. Publication bias was minimal (Table 4). High heterogeneity was observed across studies.

**Table 4:** Publication bias assessments.

Microbial measure	Begg and Mazumdar rank correlation		Egger’s regression intercept test		Duval and Tweedie’s trim and fill	
	*τ*	*P-*value	Intercept	*P*-value	Observed Hedges’ g	Adjusted Hedges’ g
Shannon	0.157	0.319	2.275	0.290	-	-
Simpson	−0.500	0.221	−18.633	0.026	-	-
P_Firmicutes	0.667	0.296	29.256	0.008	-	-
P_Proteobacteria	0.667	0.296	31.228	0.114	-	-
P_Verrucomicrobia	−0.667	0.296	−7.748	0.527	-	-
G_Bacteroides	−0.167	0.734	2.067	0.887	-	-
G_Prevotella	−0.667	0.296	−12.639	0.088	-	-
G_Megamonas	−0.167	0.734	4.789	0.956	-	-

Observed and adjusted effect sizes were reported only when missing studies were identified and corrected using Duval and Tweedie’s trim and fill. Hedges’s g was presented as overall effect size [lower limit, upper limit].

## Discussion

This meta-analysis confirms three key findings: (1) Reduced GM diversity: AD patients have lower GM diversity than HC, consistent with prior research (Vogt et al., 2017; Liu et al., 2019). (2) Stage-specific alterations: *Proteobacteria* reduction is prominent in MCI, while *Firmicutes* reduction is more pronounced in AD—suggesting GM dysbiosis progresses with cognitive decline. (3) Pathogenic taxa: Elevated *Megamonas* (linked to metabolic diseases) and *Bacteroides* [inhibits microglial A*β* clearance (Wasén et al., 2024)] may contribute to AD pathology.

The present study conducted a meta-analysis with the objective of comparing GM abundance between patients with AD spectrum and HC, and yielded three major insights into the nature of GM alterations in AD spectrum. First, patients diagnosed with AD demonstrated a reduction in GM diversity when compared to HC. Second, the microbiota present in AD and MCI remain the subject of considerable research, with a paucity of consistent findings across studies. Finally, the abundance of *Bacteroides* and *Megamonas* was progressively increased from HC to AD stage, while the abundance of *Firmicutes* and *Proteobacteria* was gradually reduced from HC to AD stage.

A plethora of studies have indicated that alpha diversity is significantly reduced in patients with AD (Vogt et al., 2017; Liu et al., 2019), yet this phenomenon has not been observed in those with MCI (Nagpal et al., 2019; Liu et al., 2021). These findings contrast with the results of our meta-analysis, which also reveal a significant reduction in MCI, in addition to a progressive decline from MCI to AD.

The *Bacteroides* is a major genus of gram-negative bacteria. It is noteworthy that prior studies have demonstrated that members of the *Bacteroidetes* phylum participate in the pathogenesis of AD by inhibiting the phagocytic function of microglia, leading to impaired β-amyloid clearance and the accumulation of amyloid plaques (Wasén et al., 2024). Despite the absence of a clearly delineated category, the text provides a modicum of direction.

A previous association has been identified between *Megamonas* and Pre-DM (Zhang et al., 2013), gestational DM (Kuang et al., 2017), and obesity (Chen et al., 2020). It is hypothesized that *Megamonas* may contribute to cerebral Aβ deposition via its role in metabolic diseases, given that both midlife obesity and DM are associated with increased risk of AD dementia or increased AD pathology.

Recent clinical trials have indicated that subjects diagnosed with MCI exhibit a substantially elevated prevalence of *Prevotella* species in their gut microbiota when compared with cognitively healthy individuals. This finding identifies specific gut microbial taxa associated with cognitive function in middle-aged and elderly populations. Should these findings be replicated, these taxonomic groups may serve as key early indicators of MCI and facilitate successful cognitive ageing through probiotic, prebiotic, and synbiotic interventions.

Despite considerable heterogeneity, this level is within expectations given the observational designs and multifactorial influences on gut microbiota. Heterogeneity primarily stems from clinical and methodological diversity: differences in AD/MCI diagnostic criteria, geography and diet, 16S rRNA sequencing platforms, and bioinformatics pipelines. Notably, all studies showed consistent effect directions, demonstrating progressive dysbiosis along the HC–MCI–AD continuum. Meta-regression and sensitivity analyses confirmed that no single study disproportionately influenced the pooled estimates. Thus, while precise effect magnitudes require further quantification, the qualitative conclusion—progressive dysbiosis accompanies cognitive decline—is robust. This meta-analysis informs future longitudinal studies and intervention strategies.

Notwithstanding the findings of the present study, there are certain limitations that must be acknowledged. Firstly, small sample sizes and geographic variability limit generalizability. Secondly, High heterogeneity due to differences in diet, clinical settings, and AD inclusion criteria. In the future, larger cohort studies are needed to validate stage-specific GM alterations and explore therapeutic targets (e.g., probiotics targeting *Firmicutes* or *Proteobacteria*).

## Data Availability

The original contributions presented in the study are included in the article/Supplementary material, further inquiries can be directed to the corresponding authors.
