# Supplementary material for: The correlation and gut microbial characteristics in the whole spectrum of Alzheimer’s disease: a systematic review and meta-analysis
Source: Front Neurosci. 2026 Mar 4;20:1775002. doi: 10.3389/fnins.2026.1775002 (PMC12996169; doi:10.3389/fnins.2026.1775002)
Supplement: Supplementary file 1 [file Data_Sheet_1.docx]

**Supplementary Material 1: Search strategies in PubMed，Web of science, Embase and MEDLINE**

**Embase, MEDLINE**

# 1 AD

# 2 Alzheimer Disease

# 3 Alzheimers Disease

# 4 Dementia

# 5 Alzheimer Dementia

# 6 Senile dementia

# 7 Senile Dementia Alzheimer Type

# 8 Presenile dementia

# 9 Primary Progressive Aphasia

# 10 Familial Alzheimer Disease

# 11 Early-Onset Alzheimer Disease

# 12 Late-Onset Alzheimer Disease

# 13 Neurodegenerative Diseases

# 14 Cognitive Dysfunction

# 15 Tau Proteins

# 16 Amyloid beta-Peptides

# 17 Plaque, Amyloid

# 18 Neurofibrillary Tangles

# 19 (#1 OR #2 OR #3 OR #4 OR #5 OR #6 OR #7 OR #8 OR #9 OR #10 OR #11 OR #12 OR #13 OR #14 OR #15 OR #16 OR #17 OR #18)

(AD) OR (Alzheimer Disease) OR (Alzheimers Disease) OR (Dementia) OR (Alzheimer Dementia) OR (Senile dementia) OR (Senile Dementia Alzheimer Type) OR (Presenile dementia) OR (Primary Progressive Aphasia) OR (Familial Alzheimer Disease) OR (Early-Onset Alzheimer Disease) OR (Late-Onset Alzheimer Disease) OR (Neurodegenerative Diseases) OR (Cognitive Dysfunction) OR (Tau Proteins) OR (Amyloid beta-Peptides) OR (Plaque, Amyloid) OR (Neurofibrillary Tangles)

# 20 Microbiome, Gastrointestinal

# 21 Gut Microbiome

# 22 Gut Microbiomes

# 23 Microbiome, Gut

# 24 Gut Microflora

# 25 Microflora, Gut

# 26 Gut Microbiota

# 27 Gut Microbiotas

# 28 Microbiota, Gut

# 29 Gastrointestinal Flora

# 30 Flora, Gastrointestinal

# 31 Gut Flora

# 32 Flora, Gut

# 33 Gastrointestinal Microbiota

# 34 Gastrointestinal Microbiotas

# 35 Microbiota, Gastrointestinal

# 36 Gastrointestinal Microbial Community

# 37 Gastrointestinal Microbial Communities

# 38 Microbial Community, Gastrointestinal

# 39 Gastrointestinal Microflora

# 40 Microflora, Gastrointestinal

# 41 Gastric Microbiome

# 42 Gastric Microbiomes

# 43 Microbiome, Gastric

# 44 Intestinal Microbiome

# 45 Intestinal Microbiomes

# 46 Microbiome, Intestinal

# 47 Intestinal Microbiota

# 48 Intestinal Microbiotas

# 49 Microbiota, Intestinal

# 50 Intestinal Microflora

# 51 Microflora, Intestinal

# 52 Intestinal Flora

# 53 Flora, Intestinal

# 54 Enteric Bacteria

# 55 Bacteria, Enteric

# 56 (#20 OR #21 OR #22 OR #23 OR #24 OR #25 OR #26 OR #27 OR #28 OR #29 OR #30 OR #31 OR #32 OR #33 OR #34 OR #35 OR #36 OR #37 OR #38 OR #39 OR #40 OR #41 OR #42 OR #43 OR #44 OR #45 OR #46 OR #47 OR #48 OR #49 OR #50 OR #51 OR #52 OR #53 OR #54 OR #55)

(Microbiome, Gastrointestinal) OR (Gut Microbiome) OR (Gut Microbiomes) OR (Microbiome, Gut) OR (Gut Microflora) OR (Microflora, Gut) OR (Gut Microbiota) OR (Gut Microbiotas) OR (Microbiota, Gut) OR (Gastrointestinal Flora) OR (Flora, Gastrointestinal) OR (Gut Flora) OR (Flora, Gut) OR (Gastrointestinal Microbiota) OR (Gastrointestinal Microbiotas) OR (Microbiota, Gastrointestinal) OR (Gastrointestinal Microbial Community) OR (Gastrointestinal Microbial Communities) OR (Microbial Community, Gastrointestinal) OR (Gastrointestinal Microflora) OR (Microflora, Gastrointestinal) OR (Gastric Microbiome) OR (Gastric Microbiomes) OR (Microbiome, Gastric) OR (Intestinal Microbiome) OR (Intestinal Microbiomes) OR (Microbiome, Intestinal) OR (Intestinal Microbiota) OR (Intestinal Microbiotas) OR (Microbiota, Intestinal) OR (Intestinal Microflora) OR (Microflora, Intestinal) OR (Intestinal Flora) OR (Flora, Intestinal) OR (Enteric Bacteria) OR (Bacteria, Enteric)

# 58 (#19) AND (#56)

((AD) OR (Alzheimer Disease) OR (Alzheimers Disease) OR (Dementia) OR (Alzheimer Dementia) OR (Senile dementia) OR (Senile Dementia Alzheimer Type) OR (Presenile dementia) OR (Primary Progressive Aphasia) OR (Familial Alzheimer Disease) OR (Early-Onset Alzheimer Disease) OR (Late-Onset Alzheimer Disease) OR (Neurodegenerative Diseases) OR (Cognitive Dysfunction) OR (Tau Proteins) OR (Amyloid beta-Peptides) OR (Plaque, Amyloid) OR (Neurofibrillary Tangles)) AND ((Microbiome, Gastrointestinal) OR (Gut Microbiome) OR (Gut Microbiomes) OR (Microbiome, Gut) OR (Gut Microflora) OR (Microflora, Gut) OR (Gut Microbiota) OR (Gut Microbiotas) OR (Microbiota, Gut) OR (Gastrointestinal Flora) OR (Flora, Gastrointestinal) OR (Gut Flora) OR (Flora, Gut) OR (Gastrointestinal Microbiota) OR (Gastrointestinal Microbiotas) OR (Microbiota, Gastrointestinal) OR (Gastrointestinal Microbial Community) OR (Gastrointestinal Microbial Communities) OR (Microbial Community, Gastrointestinal) OR (Gastrointestinal Microflora) OR (Microflora, Gastrointestinal) OR (Gastric Microbiome) OR (Gastric Microbiomes) OR (Microbiome, Gastric) OR (Intestinal Microbiome) OR (Intestinal Microbiomes) OR (Microbiome, Intestinal) OR (Intestinal Microbiota) OR (Intestinal Microbiotas) OR (Microbiota, Intestinal) OR (Intestinal Microflora) OR (Microflora, Intestinal) OR (Intestinal Flora) OR (Flora, Intestinal) OR (Enteric Bacteria) OR (Bacteria, Enteric))

**PubMed**

((AD[Title/Abstract]) OR (Alzheimer Disease[Title/Abstract]) OR (Alzheimers Disease[Title/Abstract]) OR (Dementia[Title/Abstract]) OR (Alzheimer Dementia[Title/Abstract]) OR (Senile dementia[Title/Abstract]) OR (Senile Dementia Alzheimer Type[Title/Abstract]) OR (Presenile dementia[Title/Abstract]) OR (Primary Progressive Aphasia[Title/Abstract]) OR (Familial Alzheimer Disease[Title/Abstract]) OR (Early-Onset Alzheimer Disease[Title/Abstract]) OR (Late-Onset Alzheimer Disease[Title/Abstract]) OR (Neurodegenerative Diseases[Title/Abstract]) OR (Cognitive Dysfunction[Title/Abstract]) OR (Tau Proteins[Title/Abstract]) OR (Amyloid beta-Peptides[Title/Abstract]) OR (Plaque, Amyloid[Title/Abstract]) OR (Neurofibrillary Tangles[Title/Abstract])) AND ((Microbiome, Gastrointestinal[Title/Abstract]) OR (Gut Microbiome[Title/Abstract]) OR (Gut Microbiomes[Title/Abstract]) OR (Microbiome, Gut[Title/Abstract]) OR (Gut Microflora[Title/Abstract]) OR (Microflora, Gut[Title/Abstract]) OR (Gut Microbiota[Title/Abstract]) OR (Gut Microbiotas[Title/Abstract]) OR (Microbiota, Gut[Title/Abstract]) OR (Gastrointestinal Flora[Title/Abstract]) OR (Flora, Gastrointestinal[Title/Abstract]) OR (Gut Flora[Title/Abstract]) OR (Flora, Gut[Title/Abstract]) OR (Gastrointestinal Microbiota[Title/Abstract]) OR (Gastrointestinal Microbiotas[Title/Abstract]) OR (Microbiota, Gastrointestinal[Title/Abstract]) OR (Gastrointestinal Microbial Community[Title/Abstract]) OR (Gastrointestinal Microbial Communities[Title/Abstract]) OR (Microbial Community, Gastrointestinal[Title/Abstract]) OR (Gastrointestinal Microflora[Title/Abstract]) OR (Microflora, Gastrointestinal[Title/Abstract]) OR (Gastric Microbiome[Title/Abstract]) OR (Gastric Microbiomes[Title/Abstract]) OR (Microbiome, Gastric[Title/Abstract]) OR (Intestinal Microbiome[Title/Abstract]) OR (Intestinal Microbiomes[Title/Abstract]) OR (Microbiome, Intestinal[Title/Abstract]) OR (Intestinal Microbiota[Title/Abstract]) OR (Intestinal Microbiotas[Title/Abstract]) OR (Microbiota, Intestinal[Title/Abstract]) OR (Intestinal Microflora[Title/Abstract]) OR (Microflora, Intestinal[Title/Abstract]) OR (Intestinal Flora[Title/Abstract]) OR (Flora, Intestinal[Title/Abstract]) OR (Enteric Bacteria[Title/Abstract]) OR (Bacteria, Enteric[Title/Abstract]))

**Web of science**

("AD" OR "Alzheimer Disease" OR "Alzheimers Disease" OR "Dementia" OR "Alzheimer Dementia" OR "Senile dementia" OR "Senile Dementia Alzheimer Type" OR "Presenile dementia" OR "Primary Progressive Aphasia" OR "Familial Alzheimer Disease" OR "Early-Onset Alzheimer Disease" OR "Late-Onset Alzheimer Disease" OR "Neurodegenerative Diseases" OR "Cognitive Dysfunction" OR "Tau Proteins" OR "Amyloid beta-Peptides" OR "Plaque Amyloid" OR "Neurofibrillary Tangles") AND ("Microbiome Gastrointestinal" OR "Gut Microbiome" OR "Gut Microbiomes" OR "Microbiome Gut" OR "Gut Microflora" OR "Microflora Gut" OR "Gut Microbiota" OR "Gut Microbiotas" OR "Microbiota Gut" OR "Gastrointestinal Flora" OR "Flora Gastrointestinal" OR "Gut Flora" OR "Flora Gut" OR "Gastrointestinal Microbiota" OR "Gastrointestinal Microbiotas" OR "Microbiota Gastrointestinal" OR "Gastrointestinal Microbial Community" OR "Gastrointestinal Microbial Communities" OR "Microbial Community Gastrointestinal" OR "Gastrointestinal Microflora" OR "Microflora Gastrointestinal" OR "Gastric Microbiome" OR "Gastric Microbiomes" OR "Microbiome Gastric" OR "Intestinal Microbiome" OR "Intestinal Microbiomes" OR "Microbiome Intestinal" OR "Intestinal Microbiota" OR "Intestinal Microbiotas" OR "Microbiota Intestinal" OR "Intestinal Microflora" OR "Microflora Intestinal" OR "Intestinal Flora" OR "Flora Intestinal" OR "Enteric Bacteria" OR "Bacteria Enteric")
